# Assessment of nutritional status and health-related quality of life before and after liver transplantation

**DOI:** 10.1186/s12876-015-0232-3

**Published:** 2015-01-22

**Authors:** María Teresa García-Rodríguez, María del Carmen Piñón-Villar, Beatriz López-Calviño, Alejandra Otero-Ferreiro, Francisco Suárez-López, Manuel Gómez-Gutiérrez, Salvador Pita-Fernández

**Affiliations:** 1 Digestive Service, Instituto de Investigación Biomédica de A Coruña (INIBIC), Complexo Hospitalario Universitario de A Coruña (CHUAC), Xerencia de Xestión Integrada de A Coruña, SERGAS, Universidade da Coruña, Xubias de Arriba, 84, 15006 A Coruña, Spain; 2 Clinical Epidemiology and Biostatistics Research Group, Instituto de Investigación Biomédica de A Coruña (INIBIC), Complexo Hospitalario Universitario de A Coruña (CHUAC), SERGAS, Universidade da Coruña, 15006 A Coruña, Spain; 3 Transplant Coordination, Instituto de Investigación Biomédica de A Coruña (INIBIC), Complexo Hospitalario Universitario A Coruña (CHUAC), SERGAS, Universidade da Coruña, 15006 A Coruña, Spain

**Keywords:** Liver transplantation, Nutrition status, Anxiety, Quality of life, Dependence

## Abstract

**Background:**

Patients with chronic liver disease frequently suffer from malnutrition, together with a decline in their health-related quality of life.

This study was carried out with the aim of evaluating the nutritional status, complications of medical and surgical care, anxiety, health-related quality of life and dependence level on basic and instrumental activities of daily living in pre- and post-liver transplant patients.

**Methods/Design:**

A prospective observational study with follow-up of patients on the waiting list for liver transplants who subsequently received a transplant at the University Hospital Complex in A Coruña during the period 2012–2014 (n = 110).

All the patients will be followed-up for a maximum of 6 months. For survivors, assessments will be re-evaluated at one, three and six months post- transplant.

Informed consent of the patient and ethical review board approval was obtained (Code: 2010/081 and 2010/082).

The following variables will be studied: socio-demographic data, reason for the transplant, comorbidity (Charlson Score), analytical parameters, time on transplant waiting list and post-transplant complications. A trained nurse will evaluate the following for each patient: nutritional indices, anthropometric variables and handgrip strength. Validated questionnaires will be used to determine the patients’ nutritional status (Subjective Global Assessment), anxiety (STAI questionnaire), Health-Related Quality of Life (LDQoL 1.0 questionnaire), dependence (Barthel Index and Lawton-Brody Scale), nursing diagnoses (NANDA) and post-transplant quality indicators.

Multiple linear/logistic regression models will be used to identify variables associated with the events of interest. Changes in nutritional status, quality of life and dependence over time will be analysed with linear mixed-effects regression models.

Actuarial survival analysis using Kaplan-Meier curves, Cox regression and competitive risk will be performed

Concordance between the different scores that assess nutritional status and interobserver agreement regarding nursing diagnoses will be studied using the statistical Kappa index and Bland Altman method.

**Discussion:**

The risk of malnutrition can be considered as a possible prognostic factor in transplant outcomes, associated with anxiety, health-related quality of life and dependence.

For this reason we consider interesting to perform a prospective follow-up study of patients who require a transplant to survive, studying their nutritional status and health-related quality of life.

**Electronic supplementary material:**

The online version of this article (doi:10.1186/s12876-015-0232-3) contains supplementary material, which is available to authorized users.

## Background

The liver has a metabolic function, and when affected by disease may lead to nutritional deficiency status, with liver disease patients usually suffering from Protein-Energy Malnutrition (PEM). Its appearance is caused by different factors, which include inadequate food intake, abnormal nutrient metabolism and altered digestion and absorption, together with an increased catabolism and an increase in protein-energy requirements [1-3].

The prevalence of malnutrition ranges from 25% to 80% depending on the severity of the illness and the method used to evaluate it [4,5]. In patients with decompensated cirrhosis, malnutrition varies between 60% to 100% and 20% in patients with compensated cirrhosis [6].

Malnutrition is associated with progressive liver failure, as a result of which the most malnourished patients have a worse prognosis for the illness, as morbidity and mortality increases both before and after the transplant [4,7]. It is associated with increased hospital admissions and longer stays, raising costs both before and after the transplant [5,8].

There is no consensus among authors as to which are the most effective methods for assessing the nutritional status of these patients, as the disease itself affects the values obtained [4,6]. The most frequently used methods include anthropometric parameters such as weight and height in order to calculate the patient’s Body Mass Index (BMI), Mid-Arm Circumference (MAC) and Triceps Skinfold Thickness (TSF) to calculate the Arm Muscle Circumference (AMC), the Arm Muscle Area (AMA) and Arm Fat Area (AFA) [1,2,5]. Analytical parameters such as albumin, prealbumin, transferrin, total lymphocyte, total cholesterol, creatinine and proteins [2] help to identify malnutrition and liver dysfunction [1,6].

Other methods used to assess the nutritional status in cirrhotic patients are the Subjective Global Assessment (SGA) [1,9] as well as handgrip strength, assessed with a handgrip dynamometer [6,10,11]. Bioelectrical impedance analysis [12,13] is also used, despite the fact that some authors do not consider it very useful in patients with oedema and ascites [4,6], or carrying out dietary assessments using tools such as the Malnutrition Universal Screening Tool (MUST) or Nutritional Risk Screening 2002 (NRS-2002) [4].

According to a number of studies, patients who are candidates for liver transplants have a lower perception of their Health-Related Quality of Life (HRQoL) than the general population, which varies depending on the underlying aetiology of the disease, being poorer in patients affected by hepatocellular carcinomas with alcoholic and viral liver diseases [14-17].

The facets associated with HRQoL perceived as causing the greatest concern are social isolation, depression and anxiety, sexual activity, tiredness and fatigue, gastrointestinal symptoms and pain. Fatigue is a symptom presented by 65% - 85% of the patients, the vast majority of whom perceive it as one of the symptoms that is tolerated the worst, causing incapacity to 25%. Generally these are symptoms that are minimised or not considered relevant in the progress of the illness, although they do cause anxiety to patients and can worsen their functional status [18-20].

The HRQoL is studied in relation to the progress of the patient prior and subsequent to the liver transplant.

Various instruments exist that can be used to evaluate the health-related quality of life. Amongst the generic instruments, the one considered to be the most relevant and most widely used by different authors is the Medical Outcomes Study Short Form-36 (SF-36) questionnaire, which consists of 36 items split into 8 sections, covering both physical and mental aspects [21-24].

Depending on the pathology being studied, specific questionnaires are used to evaluate its different symptoms and signs. In the case of liver disease and subsequent liver transplantation, the most usual validated questionnaire is the National Institute of Diabetes and Digestive and Kidney Disease Liver Transplant Database Quality of Life (NIDDK QOL) [23], which consists of 63 items split into 6 domains, covering aspects of social life and physical capacity. The Chronic Liver Disease questionnaire (CLDQ) [22,23] is more specific and consists of 29 items split into 6 domains, which has been validated for the Spanish population by Ferrer et al. The specific questionnaire for liver disease and subsequent liver transplantation, the Liver Disease Quality Of Life (LDQOL) [18-20] questionnaire consists of 112 items divided into 20 domains and has been validated for the Spanish population by Casanovas et al. This questionnaire has two different parts: a generic part which is equivalent to the SF-36 questionnaire [21] and a specific part on the characteristics of the disease. The LDQOL is frequently used as it contains a summarised version to evaluate the specific problems of the study population.

Also, in order to provide more data on the health-related quality of life of these patients, scales and indices will be used that measure the effect on their dependence in basic and instrumental activities of daily living (the Barthel Index and Lawton-Brody Scale) [25-28].

During the process of the disease and subsequent post-transplant recovery period, the patient may suffer from alterations to their mood and anxiety that may in turn affect the outcome of the disease [29].

Survival rates vary after liver transplants based on the level of liver dysfunction, which is measured using the Child-Pugh scales and MELD validated for this purpose. Both scales are used to evaluate liver damage, and the scores obtained are correlated with post-transplant survival [30]. Not only the degree of liver dysfunction should be considered as a prognostic factor, but factors such as the comorbidity (Charlson Score), risk of malnutrition, quality of life, dependence and anxiety can also modify the outcome of the disease.

Evaluating the patient’s nutritional status could reduce the appearance of complications before and after liver transplants, improving their health-related quality of life and reducing their dependence in basic and instrumental activities of daily living [7,12].

We consider it to be of interest to carry out a prospective follow-up study of patients affected by chronic liver disease requiring a liver transplant to survive, study their nutritional status and health-related quality of life in a reference hospital for liver transplants at national level.

Objectives:

1. To evaluate the nutritional status of patients before and after liver transplantation at one, three and six months post-surgery, and its connection with complications of surgical and medical care.
2. To determine the health-related quality of life and level of dependence in basic and instrumental activities of daily living in patients with advanced chronic liver disease on the pre-transplant list and subsequently post-transplant at one, three and six months.

Secondary objectives:

1. To evaluate their state-trait anxiety before receiving a liver transplant and six months after receiving the transplant.
2. To determine the complications and quality indicators of post-liver transplant as described by the Spanish Liver Transplant Society (SETH)
3. To determine the concordance of the nursing diagnoses (NANDA) made by two independent observers

## Methods/Design

This is a prospective observational study with follow-up, including patients on the waiting list for a liver transplant at the University Hospital Complex in A Coruña (a tertiary level 1,400-bed hospital serving a population of 516,000 in A Coruña, northwest Spain), between January 2012 and December 2014, with a minimum follow-up period of 6 months.

The study will include patients over the age of 18 who join the waiting list for a liver transplant during the study period, and who give their informed consent to take part.

The study will exclude patients whose general condition means it is impossible to make a correct evaluation, as well as those who decide not to take part on their own initiative.

### Data collection

All of the variables that will be recorded for each of the patients included in the study are summarized in Table 1 and Additional file 1: Table S2.

**Table 1:** **Data collection during the follow-up**

Variables	Base line	After Transplant		
		1 month	3 month	6 month
**Patient identification**	X			
**Comorbidity**	X			X
**Degree of hepatic dysfunction**	X			
**Anxiety assessment**	X			X
**Screening tool for Nutritional assessment**	X	X	X	X
**Analytical parametres**	X	X	X	X
**Health-related Quality Of Life (HRQoL)**	X	X	X	X
**Dependence**	X	X	X	X
**Nursing diagnoses**	X	X	X	X
**Complications after liver transplant**		X	X	X
**Liver transplant quality indicator (SETH):**				
• Post-liver transplant in-hospital mortality			X	X
• Perioperatory mortality				X
• Rate of liver retransplantation			X	X
• Rate of early reintervention			X	X

After accepting and signing the informed consent form, the patient will be identified with a code that makes it possible to keep their personal data confidential. In order to obtain the necessary information for the study, their clinical records will be studied and the patient will be interviewed, including self-administered questionnaires and a physical examination.

The patients’ clinical records will be used to compile demographic data and how long they have been on the transplant waiting list (interval in days from when they joined the list until they received the transplant). The following data will also be collected: types of pathologies leading to transplantation (alcoholic cirrhosis, autoimmune hepatitis, polycystic disease, liver cancer, primary biliary cirrhosis and viral cirrhosis), the presence of hepatic imbalances (ascites, hepatic encephalopathy, digestive bleeding, bacterial peritonitis and/or hepatorenal syndrome) and repeat transplantation. Comorbidity will be determined using the age-adjusted Charlson Score [31-34].

On joining the liver transplant waiting list and during the subsequent follow-up, the physical examination will be carried out together with the scales and validated questionnaires in order to evaluate the following:

**The degree of liver dysfunction** will be evaluated as a baseline measurement using the Child- Pugh Scale and the Model for End Stage Liver Disease (MELD), indicating that the higher the score, the greater the liver dysfunction. The Child-Pugh Scale determines the prognosis and need for transplantation, estimating the degree of liver dysfunction in these patients. The patients’ clinical records will be used to obtain the analytical parameters (serum albumin, bilirubin and prothrombin) and to identify the presence of liver imbalances (ascites and encephalopathy), obtaining a score ranging from 5 – 15 points [35,36]. MELD indicates the severity of hepatic cirrhosis and is used to prioritise patients on the waiting list for liver transplants. Based on the laboratory parameters for total bilirubin, the International Normalized Ratio (INR) and plasma creatinine, a score interval of 6–40 points is obtained [37].

**The patients’ anxiety level** will be measured at the baseline moment and then 6 months post-transplant using the State Trait Anxiety Inventory questionnaire (STAI), which will be self-administered without any time limit to complete it [38]. This questionnaire consists of two scales that measure the State Anxiety (S/A), which reflects what the patient feels at the moment of completing the questionnaire, and the Trait Anxiety (T/A), which reflects how the patient generally feels. Each scale consists of 20 items, providing a score of between 0 and 60 points (where 0 represents the minimum level of anxiety and 60 the maximum level), making it possible to obtain the percentiles according to sex and age, by comparing them with the scale chart for the questionnaire.

**Nutritional status, analytical parameters, Health-Related Quality of Life (HRQoL), dependence in basic and instrumental activities of daily living and nursing diagnoses (NANDA)** will be determined at the baseline moment and during follow up at one, three and six months post-transplant.

**Nutritional status** will be evaluated based on the analytical parameters for albumin, cholesterol and total lymphocytes, using the criteria of the Spanish Society of Parenteral and Enteral Nutrition (SENPE), Controlling Nutritional Status (CONUT) and the Nutritional Risk Index (NRI) [10,39,40]. During the physical examination the patient’s weight, height and Body Mass Index (BMI) will be obtained. Other anthropometric parameters will be calculated, such as the Mid-Arm Circumference (MAC) and Triceps Skinfold Thickness (TSF) to obtain Arm Muscle Circumference (AMC), Arm Muscle Area (AMA), Arm Fat Area (AFA) and the muscle adipose index. Handgrip strength will also be calculated, assessed with a handgrip dynamometer, as this indicates the muscular fraction of the protein compartment [10,11].

The Subjective Global Assessment (SGA) [41] will be applied, which consists of two parts: a clinical record (change in weight and intake, gastro-intestinal symptoms, functional capacity and base disease) and a physical examination to evaluate the loss of fat/muscle mass, the presence of ascites or oedemas, and the presence of tongue or skin lesions.

The clinical record will also be used to obtain the **analytical parameters** evaluated to be included on the liver transplant list (haematocrit, haemoglobin, leukocytes, albumin, total bilirubin, liver enzymes, alpha-fetoprotein, urea, creatinine, sodium, potassium, glucose, prothrombin time ratio, International Normalized Ratio (INR) and Cytomegalovirus (CMV) infection. The study will also take into account cholesterol, creatinine clearance and total lymphocytes, in order to identify how the analytical liver profile can be related to the nutritional status, health-related quality of life and level of dependence [42,43].

**Health-Related Quality of Life (HRQoL)** will be evaluated using the self-administered LDQOL 1.0 questionnaire, validated for the Spanish population and specifically developed for patients with advanced liver cirrhosis, especially for those on the liver transplant waiting list [18-20], using its abbreviated version, which consists of generic and specific dimensions. Eight generic dimensions are obtained, summarised as a physical and mental component, and eleven specific dimensions which are included due to being identified as of concern for the patients, which are Symptoms of liver disease (6 items), Effects of liver disease (3 items), Concentration (2 items), Memory (2 items), Sexual functioning (3 items), Sexual problem (2 items), Sleep (5 items), Loneliness (5 items), Health distress (3 items), Hopelessness (2 items), and Stigma of liver disease (4 items) [18-20,44].

### Degree of dependence in basic and instrumental activities of daily living

The Barthel Index will be used to evaluate basic activities, which was developed to assess changes in functional independence before and after surgery and/or treatments, and to identify the amount of care required. It is based on 10 observed items which are scored in increments of 5 (0, 5, 10, 15), with a maximum score of 100 and a minimum score of 0, considered as the maximum level of dependence [27]. To evaluate instrumental activities of daily living, the Lawton-Brody scale will be used [28] which makes it possible to evaluate functional activities based on eight items scored from eight to zero, considered as the maximum level of dependence [25-28].

**Nursing diagnoses,** considered as the basis for the nurse’s care plan, will be studied according to the use of NANDA’s taxonomy II (*North American Nursing Diagnosis Association*) which consists of 13 domains and 46 classes [45]. This evaluation will be carried out independently by two nurses, in order to study the interobserver concordance.

### Post-liver transplant complications and quality indicators will be studied in the follow-up

**The complications post - transplant** which will be evaluated are viral, bacterial and fungal infections and surgical wound infections; acute or chronic graft rejection; bleeding; anastomotic stenosis of the bile duct; thrombosis of the hepatic arteries and/or veins; repeat transplantation; repeat surgery and death.

**Post-liver transplant quality indicators**, described in the Consensus document of the Spanish Liver Transplantation Society [46] : Perioperative mortality (at one month), Post-liver transplant in-hospital mortality (at 3 and 6 months), Rate of early re-intervention (at 3 and 6 months) and Rate of liver re-transplantation (at 6 months).

### Sample size

Sample size is limited by both the duration of the study and the number of liver transplants per year. During the period 2012–2014, approximately n = 120 patients will receive a liver transplant. Assuming a loss to follow-up rate of 10%, a sample size of n = 107 patients is expected.

In relation with different objectives (prevalence, longitudinal data and prognosis) the sample is justified as detailed below.

### Prevalence

This sample size will allow us to estimate characteristics about nutritional status, quality of life and dependence with a precision of ±10% for a security: 95% (α = 0.05), assuming a prevalence of 50% of the variable of interest, assuming 10% losses during the follow-up.

### Longitudinal data

As quality of life will be assessed using the LDQOL 1.0 questionnaire, based on its 0–100 scoring system, and using range rule of thumb, a standard deviation of 25 points (one quarter of the range score) is assumed for the final scores for the questionnaire. Therefore, a sample size of n = 107 patients will allow us to estimate mean values with a precision of ±0.1 for a security: 95% (α = 0.05). Working with a power of 80% and an alpha value of 0.05, score differences of 4.5 points between groups of patients will be detected as statistically significant, assuming an exposure of 50% to the variable of interest.

This sample size will also make it possible to detect as statistically significant (p ≤0.05) correlation coefficients ≥0.15 among the studied variables and the questionnaire scores.

### Prognosis

This sample size will also make it possible to detect as significant, in a Cox regression model, a relative risk of 1.9 or more associated with risk of malnutrition, assuming an exposure to this possibility of 63.4% and a censored data percentage of 10% obtained from previous data; security: 95% (α = 0.05); statistical power: 80%, assuming an exposure prevalence of 63.4% malnutrition risk according to CONUT at the time of being included on the liver transplant list [47].

In terms of the censoring value, we have estimated it at 20%, as according to published data [48] the estimated post-liver transplant survival rate at six months is 80%. In this situation, the sample size required to estimate a relative risk of 1.85 or more (α = 0.05, β = 0.2) would be n = 105 patients.

### Statistical analysis

Descriptive analyses will be performed for all variables. Continuous variables will be reported using means ± standard deviations (SD) or median (interquartile range). For dichotomous/categorical variables, absolute numbers and percentages will be calculated, together with their 95% confidence intervals.

Nutritional status, quality of life related to health and dependence scores will be compared according to the patients’ characteristics and disease variables. The comparison of means will be carried out using Student’s T test, the Mann–Whitney test, analysis of ANOVA test and the Kruskall-Wallis test as appropriate. The association of qualitative variables will be carried out using Chi-square statistics. The correlation among quantitative variables will be assessed using Pearson and Spearman’s Rho correlation coefficient, due to the expected non-normal distribution of the questionnaires scores and to detect nonlinear relationships.

Evolution of nutritional status, health-related quality of life (HQRoL) and dependence before transplantation and at one, three and six months post-transplant from diagnosis will be analysed based on the change from the baseline scores for each time point. The significance of the changes will be assessed using the Wilcoxon signed-rank test. Clinical relevance will be analysed by effect size and Standard Error of Measurement (SEM). Finally, a value greater than 1 SEM will be considered as clinically significant.

As measurements of nutritional status, QOL and dependence will be made repeatedly for the same patients, we will be in a repeated measures context. Longitudinal nutritional status, QOL and dependence will be analysed with a linear mixed-effects regression model. More specifically, the relationship of these outcomes with time will be determined using a random coefficients model, which generalizes linear regression techniques to allow for repeated observations. These models take into account the correlation within observations on the same subject and allow for the inclusion of data on subjects who have only partial follow-up without imputing missing data.

Therefore, a linear random coefficient regression model will be performed, with nutritional status, QOL and dependence scores over time as the dependent variable. The connection with nutritional status, QOL, dependence and time will be modelled by including a quantitative time effect (months since from the listing for liver transplantation) as a covariate in the model, fitting patient effect and patient*time interaction as random effects. In addition to the effect of time on the nutritional status, QOL and dependence outcome measures, we will incorporate other factors into the regression models, in order to adjust for the variables of interest and potential confounding factors such as socio-demographic variables, hepatic dysfunction, comorbidity, analytical parameters, anxiety and follow-up complications.

In the multivariate analysis, multiple linear and logistic regression models will be used to identify those variables independently associated with patients’ nutritional status, quality of life (QOL) and dependence before and after transplantation. Separate regressions will be conducted for each of the three outcomes (nutritional status, quality of life, dependence). Box-Cox normalizing transformations will be used when necessary to ensure the normality assumption in the linear regression model.

Actuarial survival analysis with Kaplan-Meier curves, log-rank test and Cox regression analysis will be performed. The assumption of hazards proportionality will be assessed using different procedures: a) a log-minus log survival plot for each covariate, b) by analysis of scaled Schöenfeld residuals and c) by tests of interaction between categorized variables and time in the Cox model.

In case of violation of the hazard proportionality assumption for any of the covariates, an interaction term between the covariate and time will be included in the Cox regression model. Additionally, survival regression models using B-spline functions and U-shape graphs will be explored in order to model non-proportional hazards. These calculations will be performed by using the functions available in the survival package in R (version 2.10.0). As the Kaplan-Meier method could overestimate the incidence of the events in the follow-up, a competitive mortality risk survival analysis will also be considered for analysing disease-specific survival [49,50].

The analysis of the perioperative survival rate, post- transplant hospital survival rate, early intervention rate and repeat liver transplant rate will be carried out in a similar way to the analysis of the global survival rate.

Concordance between the different scores that assess nutritional status and interobserver agreement regarding nursing diagnoses at baseline and at 6 months post-transplant, will be studied with the statistical Kappa index and Bland Altman method.

Two-sided tests will be used, and p-values < 0.05 will be considered as statistically significant. Statistical analyses will be performed using SPSS for Windows (version 19.0, SPSS Inc., Chicago, Illinois), Stata (version 10) and R (version 2.12.2).

### Legal and ethical aspects

The study will be carried out according to the Good Clinical Practice guidelines of the Helsinki declaration, and the confidentiality of the information collected is guaranteed under current legislation. Informed consent is obtained from each patient to take part in the study and to review their clinical records. This project has been approved by the corresponding ethics review board (Clinical Research Ethical Committee of Galicia, decision 2010/081 and 2010/082).

## Discussion

The literature shows that the severity of liver dysfunction is associated with nutritional deficiencies and the presence of anxiety or the level of stress of the patient undergoing a liver transplant. Studies exist which show that more severe liver dysfunction is associated with a higher level of anxiety, which decreases post-surgery. This change in the levels of anxiety would be associated with an improvement in the patient’s health-related quality of life [51].

Because of these changes, it is important to know the nutritional status of the patient, as this is a risk factor for morbidity-mortality which is correlated with the severity of the liver dysfunction [52], both before and after the transplant [5,7]. It is not easy to know the incidence of malnutrition, as the parameters can be altered by the pathology itself [2,3], and so this is why it is necessary to use different methods in order to determine it, as there is no gold standard [2,40].

In order to evaluate the nutritional status, methods are used which are easy to apply, more economic and non-invasive, such as anthropometric and analytical parameters, and questionnaires on the patient’s normal intake. In this case it is advisable to use two or more, in order to compare them and determine the nutritional status of the patients or population groups being studied [40].

The most frequently used anthropometric parameters are the BMI, although this is not considered to be a reliable tool [2] as there may be changes in terms of body composition (such as the presence of oedemas or ascites). Anthropometric measurements of the upper limbs are more accepted, such as the Triceps Skinfold Thickness, Mid-Arm Circumference, Arm Muscle Area and Arm Fat Area [53,54], which will make it possible to obtain the muscle adipose index.

As some authors consider anthropometrics to be a method that underestimates the nutritional status of patients with liver disease [55], validated scores will also be obtained, such as the Controlling Nutritional Status (CONUT) [39], SENPE criteria and Nutritional Risk Index (NRI). These indices are calculated based on analytical parameters such as albumin (an indicator of the visceral protein levels), lymphocytes (an indicator of immunological function) and cholesterol (an indicator of malnutrition, related to the mortality of the patient), which make it possible to evaluate the patient’s nutritional status in combination with their weight or BMI. There is moderate concordance between them, according to the study of Gimeno et al. [40].

The SGA and handgrip with handgrip dynamometer will be used, as these are considered as techniques for determining nutritional status. The hand grip can also be considered as a tool that predicts the likelihood of malnourished patients from suffering complications [56].

The indicators of nutritional status may also be used as prognostic factors.

The triceps skinfold thickness (TSF) and the Mid-Arm Circumference (MAC) can be considered as independent prognostic factors, as patients with muscular depletion and/or severe or moderate fat have a lower survival rate [53].

The Controlling Nutritional Status (CONUT) is another method used by various authors which has been studied as an indicator of survival, which has proved to be a useful tool with a predictive power for 4-year post- liver transplant mortality in patients with advanced liver disease [57].

The treatment of chronic liver disease has changed and improved considerably in recent years, which means that imbalances such as gastro-intestinal bleeding, hepatorenal syndrome or bacterial peritonitis have a better prognosis, significantly increasing the survival of patients and the possibilities of undergoing the transplant in a better physical and mental status [15,44]. For this reason it is considered important to know the Health-Related Quality of Life (HRQoL) before the transplant and its outcome.

A significant improvement of the HRQoL is seen in the vast majority of patients, especially in terms of body image, self-esteem, taking part in active life and the ability to make plans [58].

The HRQoL and dependence in basic and instrumental activities of daily living include physical, mental and social aspects. These aspects have a significant impact on the success of a medical intervention, not only in terms of survival but also in terms of nursing care, as this includes aspects of daily life and social functioning [59].

Once the transplant has been carried out, the patient’s progress will depend on their initial status, as the severity of the liver dysfunction and the presence of complications are associated with a higher morbidity-mortality [4]. The most usual complications include bacterial infections, acute/chronic rejection or CMV infection [60]. There are also complications that may depend more on the aetiology of the transplant, with a higher incidence of repeat transplantation in patients with HCV than those with alcoholic hepatitis [61]. Other causes of repeat transplantation are acute/chronic rejection, hepatic artery thrombosis or graft dysfunction.

The post- transplant survival rate is estimated to be 80% after one year and 73% after five years [60], and is lower in patients who have undergone repeat transplantation: 67% after 3 years and 50% after 5 years [62].

This study will allow us to understand the connection between the nutritional status, the prognosis, quality of life and functional status in patients who have received liver transplants, as well as the evolution of their nutritional status before and after surgery.

### Limitations of the study

The selection bias determined by the inclusion/exclusion factors are the first limitation to the study, as patients will be included who meet the inclusion criteria regardless of the stage of their liver disease. A check will be made to verify if the results obtained are consistent with the literature.

The information bias is a result of the way in which the data are obtained. In order to minimise this bias, validated questionnaires will be used. To minimise the Observer or “Hawthorne” effect, self-administered questionnaires will be used, to prevent the interviewer from influencing the patient’s replies in any way.

It will be assumed that there may be information loss in relation to measuring the dynamometric variables if any of the patients have any kind of pathology or injuries to their upper limbs (such as arthritis, arthrosis or fractures), which have been taken into account when calculating the sample size.

In order to avoid any possible bias in relation to the measurements, these will be carried out by the same trained personnel.

Survivor selection bias is always present in follow-up studies with patients who live longer, unlike those that do not. The consistency of the results with different studies will provide us with information about the external validity of the results.

In order to control for confounding variables, a multivariate statistical analysis will be performed.

## Additional file

Additional file 1: Table S2. Baseline and post-transplant study measurements.
