# Supplementary material for: Assessment of nutritional status and health-related quality of life before and after liver transplantation
Source: BMC Gastroenterol. 2015 Jan 22;15:6. doi: 10.1186/s12876-015-0232-3 (PMC4310167; doi:10.1186/s12876-015-0232-3)
Supplement: Additional file 1: Table S2. — Baseline and post-transplant study measurements. [file 12876_2015_232_MOESM1_ESM.docx]

**Additional file**

**Table 2. Baseline and post-transplant study measurements**

| **Patient identification** | | | |
| --- | --- | --- | --- |
| - ***Sociodemographic variables*** | | | |
| - Age (years) - Gender | | | |
| **- *Other variables identification*** | | | |
| - Time in waitlist (time, in days, from the incorporation to the waitlist till transplant)  - If the patient have been retransplant  - Transplant’s etiology  - alcoholic cirrhosis  - autoimmune hepatitis  - polycystic  - hepatocarcinoma  - primary biliary cirrhosis  - virus cirrhosis  - Hepatic descompensations  - ascites  - portal-systemic encephalopathy  - digestive hemorrhage  - bacterial peritonitis  - liver kidney syndrome  - Other pathologies  - diabetes mellitus  - hypertension  - hyperlipaemia | | | |
| **Comorbidity** | | | |
| **-Charlson Score. Comorbidity component.** | | | |
| - - - Myocardial Infarction (1point).     - Congestive Heart Failure (1point).     - Peripheral Vascular Disease (1point).     - Cerebrovascular Disease (1point).     - Dementia (1point).     - Chronic pulmonary disease (1point).     - Connective Tissue Disease (1point).     - Peptic Ulcer Disease (1point).     - Diabetes Mellitus (1 point uncomplicated, 2 points if end‐organ damage)     - Moderate to Severe Chronic Kidney Disease (2 points)     - Hemiplegia (2 points)     - Leukemia (2 points)     - Malignant Lymphoma (2 points)     - Solid Tumor (2 points, 6 points if metastatic)     - Liver Disease (1 point mild, 3 points if moderate to severe)     - AIDS (6 points) | | | |
| **Degree of hepatic dysfunction** | | | |
| - ***Child-Pugh Scale*** (to set up the prognosis and the need of transplant) - ***Model for End Stage Liver Disease (MELD)*** (to give prioritize the patients in waitlist) | | | |
| **Anxiety assessment** | | | |
| - ***STAI questionnaire*** which has got two scales   - State Anxiety (S/A) refers to environment factors that protect from or generate anxiety  - Trait Anxiety (T/A) refers as personality factor that predisposes one to suffer from anxiety | | | |
| **Screening tool for Nutritional assessment** | | | |
| - ***Controlling nutritional status (CONUT)*** - ***SENPE criteria*** - ***Nutritional Risk Index (NRI)*** - ***Body Mass Index (BMI) (Kg/m²)*** - ***Anthropometrics parameters***   -Triceps skinfold thickness (TSF) (mm)  - Mid-Arm circumference (MAC) (cm)  - Arm muscle circumference (AMC) (cm)  - Arm muscle area (AMA) (cm²)  - Arm fat area (AFA) (cm²)  - Muscle adipose index (cm²)   - ***Handgrip strength*** assessed with the Jamar handgrip dynamometer - ***Subjective global assessment (SGA)*** | | | |
| **Analytical parameters** | | | |
| - Hematocrit (%)  - Hemoglobin (g/dl)  - Leukocytes (mm/c)  - Albumin (g/dl)  - Total bilirubin (mg/dl)  - Liver enzymes (ui/l)  - Alpha-fetoprotein (ui/ml)  - Urea (mg/dl)  - Creatinine (mg/dl) | | - Sodium (mEq/L)  - Potassium (mEq/L)  - Glucose (mg/dL)  - Prothrombim time ratio (s)  - International Normalized Ratio (s)  - Cytomegalovirus (CMV) infection (copies/mL)  -Cholesterol (mg/dL)  - Creatinine clearance (mL/min/1.73m^2^)  - Total Lymphocyte (%) | |
| **Health-related Quality Of Life (HRQoL)** | | | |
| **-Liver Disease Quality Of Life Questionnaire (LDQOL1.0)** | | | |
| - General. Physical component. - Funcion physical. - Role Physical. - Bodily Pain. - General health. | | - General. Mental component. - Vitality. - Social functioning. - Role emotional. - Mental health. | |
| - Specific. - Symptoms of liver disease. - Effects of liver disease. - Concentration. - Memory. - Health distress. - Sleep. | | - Loneliness. - Hopelessness. - Stigma of liver disease. - Sexual functioning. - Sexual problem. | |
| **Dependence** | | | |
| **-The Barthel Index: Basic activities of daily living.** | | | |
| - Feeding - Bathing - Grooming - Dressing - Bowels | | - Bladder - Toilet use - Transfers (Bed to chair and back) - Mobility (On level surfaces)   Stairs | |
| **-The Lawton-Brody Scale: Instrumental activities of daily living.** | | | |
| - Ability to use telephone - Shopping - Food preparation - Housekeeping | | - Laundry - Mode of transportation - Responsibility for own medications - Ability to handle finances | |
| **Nursing diagnoses** | | | |
| **-Diagnostics before liver transplantation** | | | |
| - - Imbalanced nutrition: less than body requirements.   - Excess fluid volumen.   - Constipation.   - Disturbed sleep pattern.   - Fatigue.   - Activity intolerance.   - Bathing self-care déficit.   - Dressing self-care déficit.   - Feeding self-care déficit. | | | - Toileting self-care déficit. - Situational low self-esteem. - Sexual dysfunction. - Anxiety. - Fear. - Impaired dentition. - Impaired oral mucous membrane. - Impaired tissue integrity. - Chronic pain. - Nausea. |
| **-Diagnostics after liver transplantation** | | | |
| - Ineffective Self-Health Management - Disturbed sleep pattern - Constipation - Delayed Surgical Recovery - Activity intolerance - Bathing self-care deficit - Dressing self-care deficit - Feeding self-care deficit - Toileting self-care deficit | - Readiness for enhanced knowledge - Deficient knowledge - Sexual dysfunction - Anxiety - Fear - Impaired oral mucous membrane - Hyperthermia - Acute pain - Readiness for enhanced comfort | | |
| **Complications after liver transplant** | | | |
| - ***Infections*** (wound, viral, bacterial and fungal)  - ***Rejection graft*** (chronic and acute)  - ***Haemorrhages***  *-*  ***Stenosis of the hepatic duct anastomosis***  *-* ***Thrombosis of hepatic veins or arteries***  *-*  ***Retransplantation***  *-*  ***Dead*** | | | |
| **Liver transplant quality indicator (SETH)** | | | |
| - ***Post-liver transplant in-hospital mortality*** - ***Perioperatory mortality*** - ***Rate of liver retransplantation*** - ***Rate of early reintervention*** | | | |
